# Supplementary material for: Mechanistic and evolutionary insights into a type V-M CRISPR–Cas effector enzyme
Source: Nat Struct Mol Biol. 2023 Jul 17;30(8):1172–82. doi: 10.1038/s41594-023-01042-3 (PMC10442227; doi:10.1038/s41594-023-01042-3)
Supplement: Supplementary file 1 — Supplementary Tables 1 and 2. [file 41594_2023_1042_MOESM1_ESM.pdf]

nature structural & molecular biology

Article

https://doi.org/10.1038/s41594-023-01042-3

Mechanistic and evolutionary insights into a type V-M CRISPR–Cas effector enzyme

In the format provided by the authors and unedited

Supplementary Information for

Mechanistic and evolutionary insights into a type V-M CRISPR-Cas effector enzyme

Satoshi N. Omura[1], Ryoya Nakagawa[1], Christian Südfeld[2], Ricardo Villegas Warren[2], Wen Y. Wu[2], Hisato Hirano[1], Charlie Laffeber[4], Tsukasa Kusakizako[1], Yoshiaki Kise[1,3], Joyce H.G. Lebbink[4,5], Yuzuru Itoh[1], John van der Oost[2,*], and Osamu Nureki[1,*]

[1] Department of Biological Sciences, Graduate School of Science, The University of Tokyo, 7-3-1 Hongo, Bunkyo-ku, Tokyo 113-0033, Japan

[2] Laboratory of Microbiology, Wageningen University and Research, Stippeneng 4, 6708 WE Wageningen, The Netherlands

[3] Curreio, The University of Tokyo, 7-3-1 Hongo, Bunkyo-ku, Tokyo 113-0033, Japan

[4] Department of Molecular Genetics, Oncode Institute, Erasmus MC Cancer Institute, Erasmus University Medical Center, 3000 CA Rotterdam, The Netherlands

[5] Department of Radiotherapy, Erasmus University Medical Center, 3000 CA Rotterdam, The Netherlands

[*] Correspondence: john.vanderoost@wur.nl, nureki@bs.s.u-tokyo.ac.jp

Table of Contents

Supplementary Table. 1 | Nucleic-acid sequences used for structural analysis.
Supplementary Table. 2 | Oligonucleotides used for biochemical analysis.

Supplementary Table 1. Nucleic-acid sequences used for structural analysis

Primers used in this study.	
Oligo	Sequence
RNA-T7-f	GGATCCTAATACGACTCACTATAGG
crRNA-r	GCCACGCGCACCTCATCTCCGTCTTGGCCTTCGCCCGCCAAGCTGGGCTATGACACCCTATAGTGAGTCGTA TTAGGATCC
Target DNA sequences used for the structural determination	
Target DNA strand	GATGGTGCCACGCGCACCTCATCTCCCAAATAGACA
Non-target DNA strand	TGTCTATTTGGGAGATGAGGTGCGCGTGGCACCATC

Supplementary Table 2. Oligonucleotides used for biochemical analysis.

Oligo ID	Sequence	Comment
BG29176	/5Phos/GGGCAATATTGCCGAGATGGA	FW for amplification of pML-1B-Cas12m2-Y141A
BG29177	agcCAGTGCCTTCTGATCTGCT	RV for amplification of pML-1B-Cas12m2-Y141A
BG29178	/5Phos/GCATCGTTTAATACAGTACTGG	FW for amplification of pML-1B-Cas12m2-W152A
BG29179	agcATACAGATCTCCATCTCG	RV for amplification of pML-1B-Cas12m2-W152A
BG29180	/5Phos/ACAGTACTGGATCACCATAA	FW for amplification of pML-1B- Cas12m2-N156A
BG29181	agcAAACGATGCCCAATACAG	RV for amplification of pML-1B-Cas12m2-N156A
BG29182	/5Phos/CTGCAGCGGCAGGCAGG	FW for amplification of pML-1B-Cas12m2-Q195A
BG29183	agcTACTGCAATTGTTCCCGATCCGTCA	RV for amplification of pML-1B-Cas12m2-Q195A
BG29184	/5Phos/CGGCAGGCAGGGGCACC	FW for amplification of pML-1B-Cas12m2-Q197A
BG29185	agcCAGCTGTACTGCAATTGTTCCCGAT	RV for amplification of pML-1B-Cas12m2-Q197A
BG29186	/5Phos/CGGGATGCAATTGCAGTTGT	FW for amplification of pML-1B-Cas12m2-R111A
BG29187	agcTGCTTGCCGGGCATCTTTT	RV for amplification of pML-1B-Cas12m2-R111A
BG29188	/5Phos/GATGCAATTGCAGTTGTAAAGGA	FW for amplification of pML-1B-Cas12m2-R112A
BG29189	agcCCGTGCTTGCCGGGCATCTTTTA	RV for amplification of pML-1B-Cas12m2-R112A
BG29190	/5Phos/AAGGCACGGTCGGATCAATTAGC	FW for amplification of pML-1B-Cas12m2-R126A and pML-1B-Cas12m2-R111A-R112A-R126A
BG29191	agcCCGTTCGGCTGCGTCATC	RV for amplification of pML-1B-Cas12m2-R126A
BG29192	agcCCGTTCGGCTGCGTCATCCTTTACAACTGCAATTGCATCagcagcTGCTTGCCGGGCATCTTTTAAC	RV for amplification of pML-1B-Cas12m2-R111A-R112A-R126A
BG30296	TACTGCTCTTCCATGACAACAATGACAGTACATACAATGGG	FW for amplification of inserts from pML-1B-Cas12m2-[x] for assembly of pCas-Cas12m2-[x]
BG30297	TACTGCTCTTCCcttCTAGGGGTTCGAGGGGGCAG	RV for amplification of inserts from pML-1B-Cas12m2-[x] for assembly of pCas-Cas12m2-[x]
BG30298	TACTGCTCTTCCtagaagcttggctgttttggc	FW for amplification of backbone from pCas-Cas12m2 for assembly of pCas-Cas12m2-[x]
BG30313	TACTGCTCTTCCCATGATGTCCTCCTgagctcgc	RV for amplification of backbone from pCas-Cas12m2 for assembly of pCas-Cas12m2-[x]
BG16529	GUGUCAUAGCCCAGCUUGGCGGGCGAAGGCCAAGACGUCGAGUGCAAAACCUUUCG	Cas12m PAM-SCNR RNA for SPR
BG27342	GUGUCAUAGCCCAGCUUGGCGGGCGAAGGCCAAGACUGGUCUUCGCAUCUUGCCGU	Cas12m NT RNA for SPR
	5'bio-CAGCTATAGTTCTCGAAAGGTTTTGCACTCGACTAAAGGACTCTATGACC	Biotinylated top strand with target site for SPR dsDNA
	5'GGTCATAGAGTCCTTTAGTCGAGTGCAAAACCTTTCGAGAACTATAGCTG	Bottom strand for SPR dsDNA
